# Noise exposure in early adulthood causes age-dependent and brain region-specific impairments in cognitive function

**DOI:** 10.3389/fnins.2022.1001686

**Published:** 2022-10-13

**Authors:** Salonee V. Patel, Courtney M. DeCarlo, Shae A. Book, Ashley L. Schormans, Shawn N. Whitehead, Brian L. Allman, Sarah H. Hayes

**Affiliations:** Department of Anatomy and Cell Biology, Schulich School of Medicine and Dentistry, University of Western Ontario, London, ON, Canada

**Keywords:** noise exposure, cognitive impairment, aging, microglia, hippocampus, striatum

## Abstract

Hearing loss is a chronic health condition that affects millions of people worldwide. In addition to age-related hearing impairment, excessive noise exposure is a leading cause of hearing loss. Beyond the devastating effects of hearing impairment itself, epidemiological studies have identified hearing loss as a major risk factor for age-related cognitive decline, including dementia. At present, we currently lack a full understanding of the brain regions and underlying molecular changes that are responsible for mediating the link between hearing loss and cognitive impairment across aging. In the present study, we exposed 6-month-old rats to an occupational-like noise (100 dB SPL, 4 h/day × 30 days) or sham exposure and investigated both hippocampal-dependent (i.e., spatial learning and memory, assessed using the Morris water maze) and striatal-dependent (i.e., visuomotor associative learning, assessed using an operant-conditioning task) cognitive function across aging at 7, 10, and 13 months of age. We also investigated brain region-specific changes in microglial expression following noise/sham exposure in order to assess the potential contribution of this cell type to noise-induced cognitive impairments. Consistent with human studies, the occupational-like noise exposure resulted in high-frequency hearing loss, evidenced by a significant increase in hearing thresholds at 20 kHz. Ultimately, our results suggest that not all higher-level cognitive tasks or their associated brain regions appear to be equally susceptible to noise-induced deficits during aging, as the occupational-like noise exposure caused an age-dependent deficit in spatial but not visuomotor associative learning, as well as altered microglial expression in the hippocampus but not the striatum. Interestingly, we found no significant relationships between spatial learning ability and the level of hearing loss or altered microglial density in the hippocampus following noise exposure, suggesting that other changes in the brain likely contribute to hippocampal-dependent cognitive dysfunction following noise exposure. Lastly, we found that a subset of younger animals also showed noise-induced deficits in spatial learning; findings which suggest that noise exposure may represent an increased risk for cognitive impairment in vulnerable subjects. Overall, our findings highlight that even a mild occupational-like noise exposure earlier in adulthood can have long lasting implications for cognitive function later in life.

## Introduction

Hearing loss is a chronic health condition affecting over 400 million people worldwide, with one of the leading causes being excessive exposure to loud noise from environmental (e.g., city traffic), recreational (e.g., loud music) and occupational insults (e.g., industry workers; military personnel) (Clark, 1991; Lie et al., 2016; World Health Organization [WHO], 2021). Given that recent large-scale epidemiological studies have identified hearing loss as a major risk factor for age-related cognitive decline, including dementia, there is a critical need to better understand the adverse effects of noise-induced hearing loss on the brain (Lin, 2011; Lin et al., 2011a,b, 2012; Gurgel et al., 2014; Fritze et al., 2016; Livingston et al., 2020). Based on recent studies in humans as well as more invasive studies in animal models, there is evidence that the negative effects of noise exposure are not restricted to the auditory system itself (Manikandan et al., 2006; Goble et al., 2009; Kraus et al., 2010; Basner et al., 2014; Li et al., 2014; Cui et al., 2015; Irgens-Hansen et al., 2015; Ruvalcaba-Delgadillo et al., 2015; Su et al., 2018; Cheng et al., 2019; Hayes et al., 2019; Stone et al., 2020; Ke et al., 2021; Osbrink et al., 2021; Molina et al., 2022). At present, however, we currently lack a full understanding of the brain regions and underlying molecular changes that are responsible for mediating the link between hearing loss and cognitive impairment across aging.

The vast majority of animal studies that have investigated noise-induced cognitive impairment have focused on characterizing deficits in hippocampal-dependent spatial learning and memory performance using the Morris water maze task shortly after noise exposure (Cui et al., 2009; Cheng et al., 2011; Chengzhi et al., 2011; Liu et al., 2016; Wang et al., 2016; Wieczerzak et al., 2021). Consequently, much less is known about whether other brain regions subserving cognitive processing are similarly vulnerable to noise-induced hearing loss, and by extension, it is unclear whether noise exposure in early adulthood differentially impacts specific cognitive domains later in life. As aging is associated with an increased reliance on the striatum to help compensate for hippocampal dysfunction (Bohbot et al., 2012; Rodgers et al., 2012; Wiener et al., 2013; Zhong and Moffat, 2018), it is important to determine how noise exposure affects the short- and long-term function of the striatum—a brain region that normally subserves stimulus-response habit-learning and goal-directed actions (McDonald et al., 2007; Delotterie et al., 2015). To date, only a few animal studies have investigated the effect of noise exposure on the striatum. For example, noise exposure can alter striatal neurotransmitter systems (e.g., dopamine, serotonin, acetylcholine, glutamate) (Ravindran et al., 2005; Sembulingam et al., 2005; Samson et al., 2006; Kazi and Oommen, 2014), and we recently found that in the weeks following intense noise exposure, rats not only exhibited deficits in hippocampal-dependent learning and memory, but also demonstrated impaired performance on a simple visual cue discrimination task largely reliant on the striatum (Wieczerzak et al., 2021). Whether more complex striatal-dependent cognitive functions, such as visuomotor associative learning, are also impaired by noise exposure has yet to be investigated. Furthermore, it is still not fully understood if hippocampal- and/or striatal-dependent cognitive impairments persist (or become exacerbated) in the months following noise exposure; findings which could go on to have significant implications for an acceleration of age-related cognitive decline.

It is well-established that noise exposure can induce inflammation in the peripheral and central auditory pathway (Henderson et al., 2006; Tornabene et al., 2006; Fuentes-Santamaria et al., 2017). In particular, noise exposure has been shown to cause an increase in the expression of microglia in both the auditory brainstem and cortex (Baizer et al., 2015; Fuentes-Santamaria et al., 2017; Wang et al., 2019; Zhuang et al., 2020). Microglia are the resident immune cells of the central nervous system and have numerous ramifications through which they search for environmental stressors [for review, see Kettenmann et al. (2011)]. Upon encountering an insult, microglia become activated and undergo rapid morphological changes, characterized by shorter ramifications and a larger soma, to provide neuroprotective effects. If this state persists, as commonly seen in aging and neurological disorders, chronically activated microglia are hypothesized to play a neurotoxic role, releasing proinflammatory cytokines, and contributing to neural degradation (Block et al., 2007). Of concern, alterations in microglial expression have also been observed in the hippocampus of noise-exposed rodents and these immune cells have been theorized to underly the cognitive impairments commonly seen following noise exposure (Zhuang et al., 2020; Li et al., 2021). However, no studies have examined if noise-induced alterations in microglial expression occur in other cognitive brain regions, such as the striatum, further contributing to impairments in cognitive function.

Previous investigations into the link between hearing loss and cognitive impairment in animal models have utilized methodological approaches that may limit their translatability for noise-induced hearing loss in humans. For example, many of these experimental studies have employed intense noise exposures, in excess of 120 dB SPL, that cause severe hearing loss or deafness; an approach that does not closely resemble the most common forms of noise-induced hearing loss resulting from environmental, recreational, and occupational settings (Kraus et al., 2010; Tao et al., 2015; Liu et al., 2016, 2018; Zhuang et al., 2020; Li et al., 2021). Furthermore, many studies have investigated cognitive function in the immediate days or weeks following noise exposure (Cui et al., 2009; Cheng et al., 2011; Wang et al., 2016), highlighting the need to determine if noise-induced cognitive impairments persist across aging. Thus, in the present study we exposed 6-month-old rats to an occupational-like noise exposure (100 dB SPL, 4 h/day × 30 days) and assessed both hippocampal- (i.e., spatial learning and memory) and striatal-dependent (i.e., visuomotor associative learning) cognitive function at 7, 10, and 13 months of age. We also investigated the brain region-specific changes in microglial expression following noise exposure in order to assess the potential contribution of this cell type to noise-induced cognitive impairments. Ultimately, our results suggest that not all higher-level cognitive tasks or their associated brain regions appear to be equally susceptible to noise-induced deficits during aging, as the occupational-like noise exposure caused an age-dependent deficit in spatial but not visuomotor associative learning, as well as altered microglial expression in the hippocampus but not the striatum. Furthermore, we found that a subset of younger animals also showed noise-induced deficits in spatial learning; findings which suggest that noise exposure may represent an increased risk for cognitive impairment in vulnerable subjects.

## Experimental procedures

### Subjects and experimental design

**FIGURE 1:**
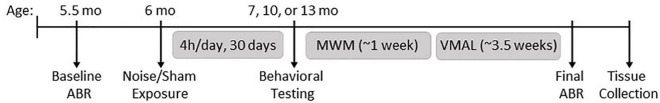
Experimental timeline. Rats underwent a baseline hearing test measured using the auditory brainstem response (ABR) at 5.5 months of age, followed by noise or sham exposure at 6 months of age (100 dB SPL; 4 h/day, 30 days). Behavioral testing began at 7 months (immediately following noise exposure), 10 or 13 months of age, and included the Morris water maze (MWM) and visuomotor associative learning tasks (VMAL). Upon completion of behavioral testing, a final hearing test was performed, and rats were then sacrificed for tissue collection.

The present study utilized Fischer 344 rats, a rodent model commonly used to study cognitive performance across the lifespan (vanderStaay and Blokland, 1996; LaSarge et al., 2007; Bizon et al., 2009). Of importance, compared to young-adult (i.e., 6-month-old) rats, age-related impairments in spatial learning and memory performance have been shown to occur by 12 months of age (i.e., older-adulthood) in this strain (Bizon et al., 2009). Thus, in order to assess both shorter and longer-term effects of noise exposure in early adulthood on cognitive function across aging, male and female Fischer 344 rats underwent noise or sham exposures at 6 months of age, with separate cohorts of rats then undergoing cognitive-behavioral testing at either 7 (Sham: 9 male, 8 female; Noise: 9 male, 12 female), 10 (Sham: 9 male, 8 female; Noise: 9 male, 9 female), or 13 (Sham: 10 male, 8 female; Noise: 10 male, 9 female) months of age (Figure 1). To ensure litter effects were not a confounding variable in our results, animals from each litter were distributed equally between treatment and age groups. Hearing sensitivity was assessed before noise or sham exposure and immediately before tissue collection. Experimenters were blinded to the identity of the animal treatment groups and age for the collection and analysis of all data. All rats used in this study were housed in facilities maintained by Animal Care and Veterinary Services on a 12-h light/dark cycle with food and water *ad libitum* (unless stated otherwise). Experimental procedures were approved by the Animal Care Committee at Western University and in compliance with the guidelines established by the Canadian Council on Animal Care.

### Hearing assessment

Hearing sensitivity was measured using an auditory brainstem response (ABR) protocol. Rats were anesthetized with isoflurane (4% induction, 2% maintenance), placed in a sound-attenuating chamber (ENV-017M; Med Associates, St. Albans, VT, USA), and maintained at a temperature of ∼37°C using a homeothermic heating pad (507220F; Harvard Apparatus, Holliston, MA, USA). Subdermal electrodes (27 gauge; Rochester Electro-Medical, Lutz, FL, USA) were positioned at the vertex (active electrode), over the right mastoid process (reference electrode), and on the midback (ground electrode).

Auditory stimuli consisting of 2 tones (4 and 20 kHz; 5 ms duration and 1 ms rise/fall time) were generated using a Tucker-Davis Technologies (TDT) RZ6 processing module with a 100 kHz sampling rate (TDT, Alachua, FL, USA) and calibrated with custom MATLAB software (The Mathworks, Natick, MA, USA) using a 1/4-inch microphone (2530; Larson Davis, Depew, NY, USA) and preamplifier (2221; Larson Davis). The auditory stimuli were delivered by a speaker (MF1; TDT) positioned 10 cm from the animal’s right ear while the left ear was occluded with a custom foam earplug. All stimuli were presented 1,000 times (21 times/s) at decreasing intensities from 90 to 10 dB sound pressure level (SPL) in 10 dB steps. Near threshold, successive steps were decreased to 5 dB SPL, and each sound level was presented twice to best determine the ABR threshold using the criteria of a just noticeable deflection of the averaged electrical activity within the 10-ms time window. Sound-evoked responses were acquired using a low-impedance headstage (RA4LI; TDT), preamplified and digitized (RA16SD Medusa preamp; TDT) and sent to an RZ6 processing module *via* a fiber optic cable. The signal was filtered (300–3,000 Hz) and averaged using BioSig software (TDT).

### Noise exposure

In an effort to replicate the intensity of repetitive noise exposures commonly experienced by individuals working in noisy environments (i.e., an occupational-like noise exposure) rats underwent noise (100 dB SPL white noise) or sham (speakers off) exposures for 4 h/day for 30 consecutive days. Both noise and sham exposures were carried out in a sound-attenuating booth (MDL 4872 S; WhisperRoom, Inc., Knoxville, TN, USA) containing a wire shelving unit (Nexel Industries Inc., Port Washington, NY, USA) from which 20 equally spaced speakers (4 speakers per row, 5 rows; PDS122; Pyle USA, Brooklyn, NY, USA) were suspended. During the exposures, animals were placed individually into modified home cages, with each cage placed directly underneath one of the suspended speakers. White noise (1–20 kHz) was generated (Audacity, version 2.3.2), amplified (XLS1000, Crown) and distributed (SP-160-10V; Specialty AV) to the speakers and calibrated to 100 dB SPL with a sound level meter (WS1361C; Koolertron) placed at the level of the rat’s ears within the cage. Rats underwent noise or sham exposures at 6 months of age, with the placement of each rat rotated throughout the booth over the 30-day exposure period to ensure that all animals had equivalent exposures.

### Morris water maze

Following noise or sham exposures, separate cohorts of rats were assessed for spatial learning and reference memory performance using the Morris water maze at either 7, 10, or 13 months of age. The maze consisted of a circular pool (144 cm diameter) filled with room-temperature water (22–23°C) that was dyed with black non-toxic acrylic paint. The pool was divided into four quadrants and given north (N), south (S), east (E), and west (W) designations to define specific points around the circular perimeter. The protocol occurred over a total of 8 days, where rats underwent 2 days of habituation, followed by 5 days of spatial learning trials, and then 1 day consisting of the probe test and visual cue trials.

On the first 2 days of the protocol, rats were habituated to the pool and the presence of a submerged platform located in the center of the pool (12 cm in diameter; 3 cm below the surface of the water). On both habituation days, rats were placed directly on the platform for 30 s. On the second day of habituation, after spending 30 s on the platform, the rats were removed, immediately placed in the north position of the pool, and allowed to swim to the platform. For the subsequent days of spatial learning and probe trials, spatial cues were placed at the northwest (red square), southwest (green triangle), northeast (white star), and southeast (yellow plus-sign) corners of the pool, and the platform was positioned in the center of the southwest quadrant. Rats underwent spatial learning trials for 5 consecutive days, with four 90-s trials (1-h inter-trial interval) per day to assess their ability to locate the submerged platform over time. On each day, rats were released facing the pool wall in the northwest (NW), north (N), northeast (NE), and east (E) positions for trials 1–4, respectively. If the rat did not find the platform within the 90-s maximum trial duration, it was cued to the platform by the experimenter, and allowed to rest on the platform for 30 s to observe its location with respect to the spatial cues. Throughout testing, each rat’s time to locate the hidden platform and their average swim speed were collected using ANYmaze tracking software (v6.33, 64-bit; 267 Stoelting Company, Wood Dale, IL, USA) and a webcam (C930e; Logitech, Switzerland) mounted on the ceiling above the maze.

On the last day of testing, the platform was removed for the probe trial, during which the rats were placed in the NNE position and allowed to swim for the full 90 s. Reference memory was assessed by the time required for the rat to recall the location of the platform region (15 cm diameter region centered on where the platform was previously placed). One hour after the completion of the probe test, visual cue trials were conducted to identify any potential differences in visual acuity and/or swim speed between the sham and noise-exposed rats, as these factors can confound the interpretation of learning trial and probe test data. The spatial cues were removed from around the pool, and the location of the platform was now indicated using a marker (flag) positioned directly on the platform and protruding from the surface of the water. Rats underwent four trials (1-h inter-trial interval) in which they were placed in the north, west, south, and east start positions of the pool with the cued-platform located in the southeast, northeast, southwest, and northwest quadrants, respectively.

### Visuomotor associative learning

Following the completion of the 8-day Morris water maze task, visuomotor associative learning (VMAL) was assessed in male rats in a standard modular test chamber (ENV-008CT; Med Associates) housed in a sound-attenuating box (29″ W by 23.5″ H by 23.5″ D, Med Associates). The behavioral chamber was illuminated by a house light on the back wall, whereas the front wall contained a center nose poke, a left feeder trough and a right feeder trough; each equipped with an infrared detector. For presentation of visual stimuli, an LED (ENV-229M; Med Associates) was located above the center nose poke and delivered either a steady light stimulus (1-s duration) or a flashing light stimulus (5 times/s, 1-s duration). Stimulus delivery, nose-poke responses and food rewards were controlled and monitored using custom behavioral protocols running in MATLAB (Epsych Toolbox1) which was interfaced with real-time processing hardware (RZ6; TDT).

Before beginning the visuomotor associative learning task, rats were food-restricted, weighed daily and maintained at >85% of their free-feeding body mass. They were then habituated to receiving sucrose pellets upon initiation of a trial by performing a nose poke in the center port. Once rats reached ∼150 trials during their daily 30-min session (∼6-day training period), the testing phase began. During the testing phase, one of two visual stimuli was presented upon the initiation of a trial by a nose poke in the center port. Over 18 consecutive days, rats learned to access the left feeder trough when the steady light stimulus was presented, and the right feeder trough when the flashing light stimulus was presented. The rats performed ∼150 trials per day. Custom MATLAB scripts were used to obtain the rats’ overall performance (percent accuracy) and time taken to respond correctly to a given stimulus (reaction time) for each day of the visuomotor associative learning task.

### Immunohistochemistry

Following the final ABR hearing assessment, noise-exposed and sham rats were deeply anesthetized with isoflurane and perfused through the heart with saline followed by 4% paraformaldehyde. Following perfusion, brains were removed and post-fixed in 4% paraformaldehyde overnight at 4°C. The following day, tissue was cryoprotected in 15% followed by 30% sucrose in phosphate buffer (PB, 0.1 M) until the tissue sank. Brain tissue was then cut into 30 μm thick coronal sections using a cryostat and stored in storage solution (30% ethylene glycol, 30% glycerol in 0.1 M PBS) at −20°C until further processing.

Immunolabeling was carried out on free-floating tissue sections. For every immunolabeling session, noise-exposed and sham tissue sections were processed in parallel using the same solutions. Tissue sections were initially removed from storage solution and rinsed in PBS. Tissue sections were washed in PBS for 30 min at room temperature between each of the following incubation steps. For deactivation of endogenous peroxidase, sections were treated with 1% H_2_O_2_ (Thermo Fisher Scientific, Waltham, MA, USA) in PBS for 10 min. Next, the sections were pre-treated with blocking solution containing 2.5% normal horse serum (VECTS2000; Vector Laboratories, Newark, CA, USA) and 0.2% Triton X-100 (X100; MilliporeSigma, Burlington, MA, USA) in PBS for 1 h. Sections were then incubated with primary antibody against Iba1 (1:1000; 019-19741; Wako Chemicals, Richmond, VA, USA) in blocking solution overnight at 4°C. Next, sections were incubated with biotinylated secondary antibody (1:500; anti-rabbit IgG, BA1100; Vector Laboratories) in blocking solution for 2 h at room temperature. Sections were processed using ABC kits (32020; Thermo Fisher Scientific) and labeling was visualized using diaminobenzidine (DAB). Immunolabeled sections were mounted on Fisher Superfrost polarized slides and dried overnight. Slides were then dehydrated in increasing concentrations of alcohol, cleared in xylene, and sealed using DPX (1005790507; MilliporeSigma).

Microglial Iba1 expression was visualized in coronal sections of the hippocampus (bregma −3.00 mm, CA1 subregion), auditory cortex (bregma −5.50 mm), and dorsal striatum (bregma 0.00 and 1.00 mm). All images were acquired using a digital camera on an upright brightfield microscope (Nikon Eclipse Ni-E, Nikon DS Fi2 color camera, NIS Elements Imaging; Mississauga, ON, USA), wherein white balance was automated using an off-tissue reference point and all settings (light intensity, exposure, aperture) were kept constant for all micrographs. Quantification of Iba1 expression was carried out using ImageJ software (version 1.53r). To assess microglial expression we quantified Iba1-positive cell morphology and density using soma-to-cell-size ratios and cell counts per micrograph, respectively. Stacked micrographs were acquired at 40× (5–8 z-plane images per stack), with four to eight randomly selected images taken within each region of interest. As previously described (Hovens et al., 2015), microglial soma area was defined as the spherical soma region of Iba1-positive microglia, while the microglial cell size was defined as the area formed by the connection of the outermost points of each dendritic process of Iba1-positive microglia. Using the polygon trace tool in ImageJ, microglial soma and cell size areas were measured for two randomly selected microglia in each 40× micrograph. Using the same 40× stacked micrographs, microglial cell number was assessed by manually counting the number of Iba1-positive microglia per micrograph. The microglial cell number for each rat was averaged among the separate 40× stacked micrographs and divided by the area of the image to obtain microglial density (cells per millimeter squared) in each region of interest. All imaging and analysis were carried out by an experimenter blinded to each rat’s treatment group and age.

### Data presentation and statistics

Statistical analyses were conducted using SPSS software (Version 27; IBM Corporation, Armonk, NY, USA), and included two-way or three-way analysis of variance (ANOVA) at each time point of age (see section “Results” for the details of each specific comparison). If Mauchly’s test of sphericity was violated within the repeated-measures ANOVA, the Greenhouse-Geisser correction was used. The level of significance was set at *a* = 0.05 and when appropriate, Bonferroni’s *post-hoc* corrections were used. GraphPad Prism (Version 9; Software Incorporated, La Jolla, CA, USA) was used to plot the graphs and BioRender (Biorender.com) was used for methodology schematics. Data are presented as mean values ± standard error of the mean (SEM).

## Results

### Noise exposure resulted in an age- and sex-dependent mild, high-frequency hearing loss

**FIGURE 2:**
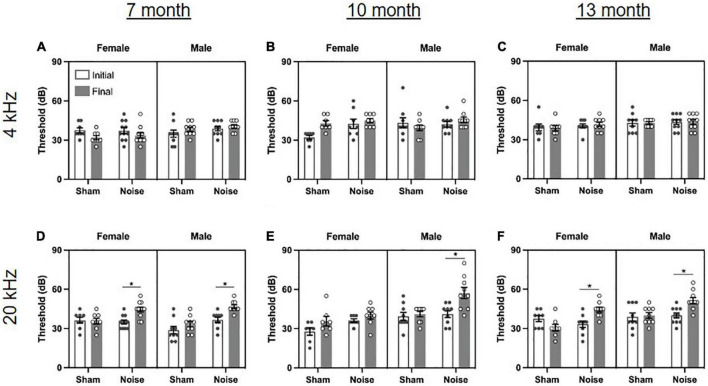
Noise exposure caused an age- and sex-dependent mild, high-frequency hearing loss. **(A–C)** The auditory brainstem response (ABR) revealed no changes in hearing sensitivity at the 4 kHz stimulus following noise or sham exposure at 7, 10, or 13 months of age. **(D,F)** Noise-exposed rats had significantly increased hearing thresholds compared to baseline at the 20 kHz stimulus at 7 and 13 months of age, regardless of sex (*p_bonf_ < 0.05). **(E)** At 10 months of age, noise-exposed male rats, but not female rats, had significantly increased hearing thresholds at the 20 kHz stimulus (*p_bonf_ < 0.05). *n* = 7-month (Sham: 9 male, 8 female; Noise: 9 male, 11 female), 10-month (Sham: 9 male, 7 female; Noise: 9 male, 8 female), and 13-month (Sham: 9 male, 8 female; Noise: 10 male, 9 female). Data represent group mean ± SEM.

To assess the effect of the occupational-like noise exposure on hearing sensitivity, the ABR thresholds before and after noise/sham exposure were evaluated at each of the tonal stimuli presented (4 kHz, 20 kHz) (Figure 2). At each time point of age, a three-way ANOVA (treatment × sex × time) for each stimulus type was performed. As expected, there were no significant differences in hearing sensitivity for rats following the sham exposure. Although the noise-exposed rats showed no changes in hearing sensitivity for the 4 kHz stimulus (Figures 2A–C), they experienced high-frequency hearing loss, as evidenced by significantly increased hearing thresholds for the 20 kHz stimulus for 7-month [treatment × time interaction: *F*_(1,33)_ = 10.543, *p* = 0.003; *p*_bonf_ < 0.001; Figure 2D] and 13-month-old rats [treatment × time interaction: *F*_(1,32)_ = 21.929, *p* < 0.001; *p*_bonf_ < 0.001; Figure 2F], regardless of sex. Furthermore, 10-month-old males [treatment × sex × time interaction: *F*_(1,29)_ = 8.975, *p* = 0.006; *p*_bonf_ < 0.001], but not 10-month-old females, had significantly increased hearing thresholds at the 20 kHz stimulus (Figure 2E). Taken together, these data show that the occupational-like noise exposure protocol induced an age- and sex-dependent mild, high-frequency hearing loss. These results are consistent with previous studies in human and animal subjects which have also found age and sex-dependent effects of noise exposure on hearing thresholds (Szanto and Ionescu, 1983; Milon et al., 2018; Nolan, 2020) and suggest that female rats may demonstrate resiliency or some recovery of hearing sensitivity (e.g., 10-month-old females, Figure 2E) following noise exposure that is not observed in male rats.

### Hippocampal-dependent spatial learning was impaired in noise-exposed 13-month-old rats and a subset of noise-exposed 10-month-old rats

**FIGURE 3:**
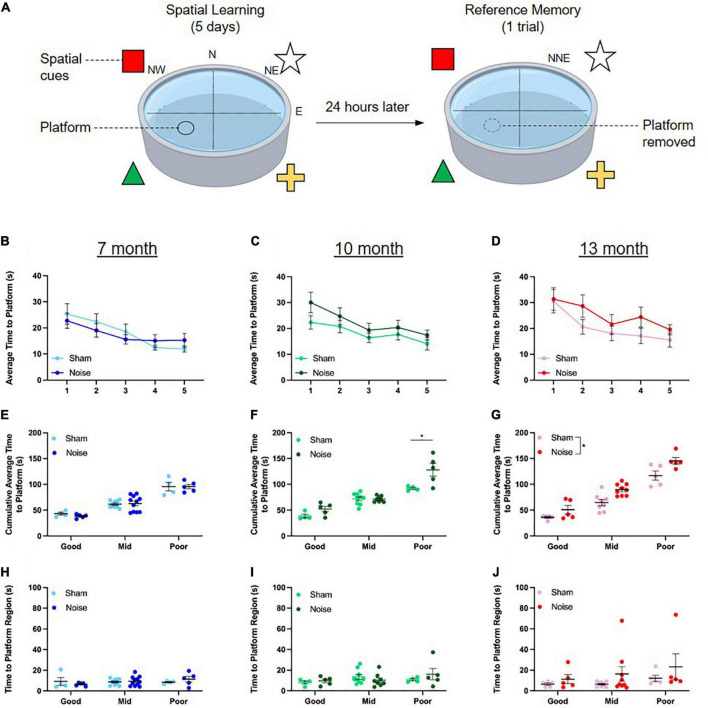
Hippocampal-dependent spatial learning was impaired in noise-exposed 13-month-old rats and a subset of noise-exposed 10-month-old rats. **(A)** During the spatial learning component of the Morris water maze task, rats underwent 4 trials/day for 5 days, utilizing extra-maze visual cues placed on the wall to locate a submerged platform in the pool. On each day, rats were released facing the pool wall in the NW, N, NE, and E positions for trials 1–4, respectively. On the 6th day, rats were placed in the NNE location and underwent one trial to assess their reference memory during which the platform was removed. **(B–D)** Rats demonstrated spatial learning, as evidenced by improved performance on the task over the 5 days of testing. There were no significant differences in the learning curves between noise- and sham-exposed rats at any age. **(E,F)** While noise exposure had no effect on the cumulative time to reach the platform at 7 months of age, spatial learning was impaired in the poorly performing noise-exposed rats at 10 months of age (*p_bonf_ < 0.05). **(G)** By 13 months of age, noise exposure impaired spatial learning, regardless of performance type (significant main effect of treatment, *p < 0.05). **(H–J)** At all ages, reference memory was unaffected by noise exposure. *n* = 7-month (Sham: 9 male, 8 female; Noise: 9 male, 12 female), 10-month (Sham: 9 male, 8 female; Noise: 9 male, 9 female), and 13-month (Sham: 10 male, 8 female; Noise: 10 male, 9 female). Data represent group mean ± SEM.

To determine the effect of noise exposure on spatial learning in the Morris water maze, each rat’s time to platform was averaged across the four learning trials for each of the 5 days of testing. The first trial on Day 1 of testing was omitted for each rat as this represents their first time navigating the pool and is not indicative of spatial learning. A three-way ANOVA (treatment × sex × day) was performed at each time point of age. As shown in the learning curves in Figures 3B–D, rats at each age demonstrated spatial learning, as evidenced by improved performance on the task over the 5 days of testing [significant main effect of day for all ages; 7 month: *F*_(4,136)_ = 6.784, *p* < 0.0001; 10-month: *F*_(4,124)_ = 5.609, *p* < 0.0001; 13-month: *F*_(4,132)_ = 6.544, *p* < 0.0001]. However, no significant differences were found between the learning curves of sham and noise-exposed rats at 7, 10, or 13 months of age. In addition to examining daily performance on the task, spatial learning was quantified by calculating each rat’s cumulative time to reach the hidden platform on the learning trials. Consistent with a past study which investigated the effect of age on spatial cognition (Young et al., 2010) and in an effort to discern whether some rats were more vulnerable to noise-induced cognitive deficits than others, the rats within each experimental group (i.e., sham or noise-exposed rats at 7, 10, or 13 months of age) were further classified as *good* (top 25th percentile), *mid* (middle 50th percentile), or *poor* (lowest 25th percentile) performers based on their cumulative time to reach the platform (Figures 3E–G). Three-way ANOVAs (treatment × sex × performer type) were conducted separately for each time point of age, and the collective results show that the effect of noise exposure varied by age and performance classification. Although noise exposure had no effect on the cumulative time to reach the hidden platform at 7 months of age (Figure 3E), it significantly impaired spatial learning in the poorest performing rats at 10 months of age [treatment × performer type interaction: *F*_(2,26)_ = 3.798, *p* = 0.036; *p*_bonf_ < 0.001; Figure 3F]. By 13 months of age, noise exposure significantly impaired spatial learning, whereby noise-exposed rats took significantly longer to reach the hidden platform compared to their sham-exposed counterparts, regardless of performer type [main effect of treatment: *F*_(1,25)_ = 12.632, *p* = 0.002; Figure 3G]. Carrying forward each animal’s performer type classification from the spatial learning analysis, spatial reference memory was assessed using the time it took for rats to first reach the previous location of the hidden platform during the probe test session. Three-way ANOVAs (treatment × sex × performer type) were conducted and revealed no significant differences at any age, indicating that spatial reference memory was unaffected by noise exposure (Figures 3H–J). The collective results from the Morris water maze show that spatial learning was more susceptible to noise exposure than spatial reference memory, and that while learning was impaired across all performers in the 13-month age group, only a subset of poorly performing rats were vulnerable to noise exposure in the 10-month age group.

### Noise-exposed rats demonstrated no significant deficits in performance accuracy on the visuomotor associative learning task, but exhibited a faster reaction time in the month post-noise exposure

**FIGURE 4:**
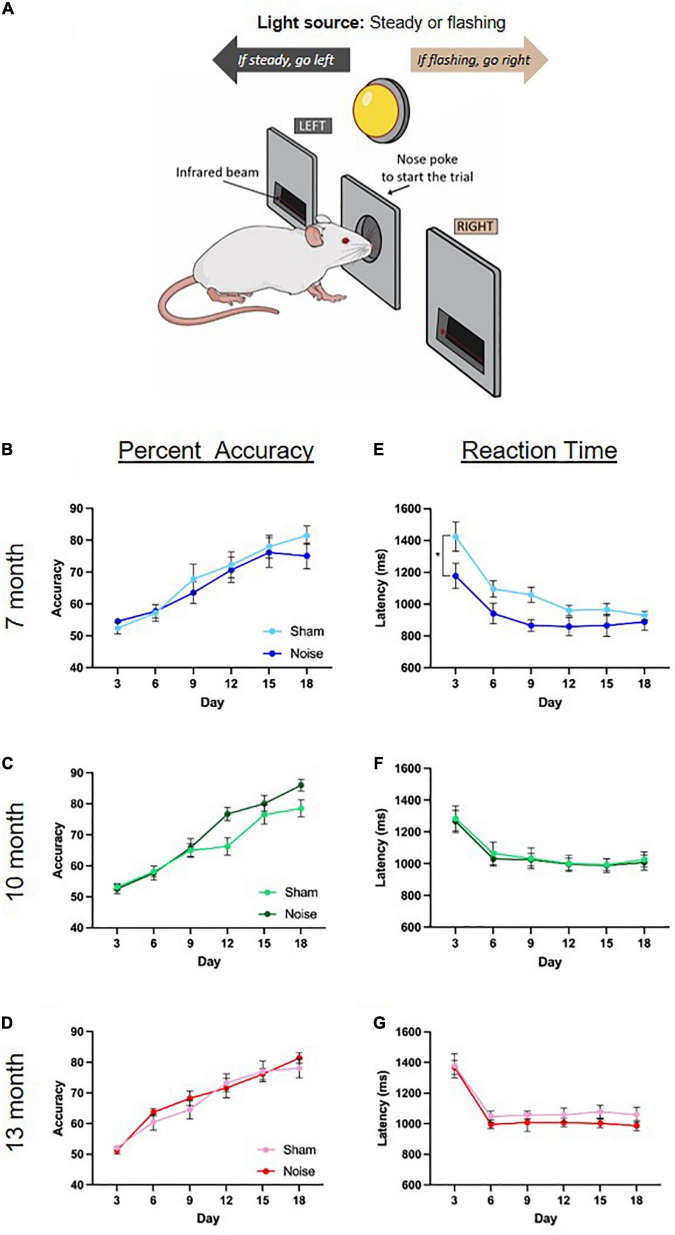
Noise-exposed rats demonstrated no significant deficits in performance accuracy on the visuomotor associative learning task, but exhibited a faster reaction time in the month post-noise exposure. **(A)** Rats were trained on an operant conditioning-based visuomotor associative learning task. Following the initiation of a trial caused by a noise poke in the center port, rats learned to access the left feeder trough when the steady light stimulus was presented, and the right feeder trough when the flashing light stimulus was presented. **(B–D)** Rats at each age demonstrated learning over the 18 days of the task, with no significant differences in percent accuracy between noise- or sham-exposed rats at any age. **(E–G)** Noise-exposed rats at 7 months of age showed a significantly faster reaction time than their sham-exposed counterparts, an effect that did not persist at 10 or 13 months of age (significant main effect of treatment, *p < 0.05). *n* = 7-month (Sham: 9 male; Noise: 9 male), 10-month (Sham: 9 male; Noise: 9 male), and 13-month (Sham: 10 male; Noise: 10 male). Data represent group mean ± SEM.

To determine the effect of noise exposure on visuomotor associative learning, male rats’ overall performance (percent accuracy) and time taken to respond correctly to a given stimulus (reaction time) were collected over an 18-day period. For both metrics, 3-day averages were calculated to obtain a total of six time points over the 18-day period (days 3, 6, 9, 12, 15, 18). To first characterize the rats’ rate of learning on the task, separate two-way ANOVAs (treatment × day) were conducted for each age group. As shown in the learning curves in Figures 4B–D, rats at each age demonstrated an ability to learn the task, as evidenced by increasing percent accuracy over 18 days of testing [significant main effect of day for all ages; 7 month: *F*_(2.65,42.31)_ = 37.240, *p* < 0.001; 10-month: *F*_(5,80)_ = 82.403, *p* < 0.001; 13-month: *F*_(3.11,55.92)_ = 65.587, *p* < 0.001]. There were no significant differences in these learning curves between noise- and sham-exposed animals at 7, 10, or 13 months of age. Next, the effect of noise exposure on reaction time was assessed at each time point of age using two-way ANOVAs (treatment × day). Noise-exposed rats at 7 months of age showed a significantly faster reaction time than their sham-exposed counterparts [main effect of treatment: *F*_(1,16)_ = 6.315, *p* = 0.023; Figure 4E], whereas there were no significant differences found in the 10-month and 13-month-old rats (Figures 4F,G). Taken together, the results from the 18-day visuomotor associative learning task show no effect of occupational-like noise exposure on the rats’ overall accuracy, but a faster reaction time in the first few weeks post-noise exposure.

### Noise exposure altered microglial density in the hippocampus, but not the auditory cortex or striatum

**FIGURE 5:**
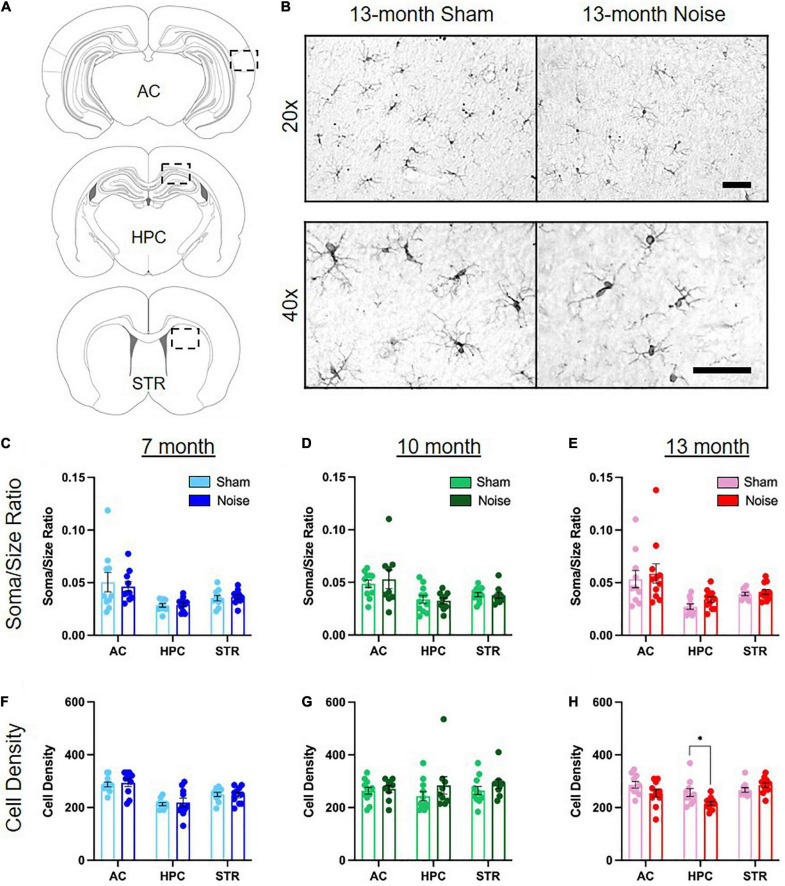
Noise exposure altered microglial density in the hippocampus, but not the auditory cortex or striatum. **(A)** Coronal section and outline for the auditory cortex (AC), hippocampal CA1 (HPC), and striatum (STR) regions of interest. **(B)** Representative 20× (top row) and 40× (bottom row) images of Iba1-positive microglia in the hippocampal CA1 of 13-month sham- and noise-exposed rats. Further quantification was performed on 40× images. See Supplementary Figure 1 for additional images. Scale bar = 50 μm. **(C–E)** There was no difference in Iba-1-positive soma/size ratios between sham- and noise-exposed rats in the auditory cortex, hippocampus, or striatum at any age. **(F,G)** Microglial cell density was not affected by noise exposure in any brain region in 7- and 10-month-old animals. **(H)** There was a significant decrease in microglial cell density in the hippocampus in noise-exposed rats at 13 months of age, but not in the auditory cortex or striatum (*p_bonf_ < 0.05). *n* = 7-month (Sham: 4 male, 6 female; Noise: 5 male, 5 female), 10-month (Sham: 6 male, 5 female; Noise: 4 male, 5 female), and 13-month (Sham: 6 male, 4 female; Noise: 6 male, 5 female). Data represent group mean ± SEM.

Previous studies have implicated a role for altered microglial expression in noise-induced brain pathology in the auditory pathway (Baizer et al., 2015; Fuentes-Santamaria et al., 2017; Wang et al., 2019) and hippocampus (Cui et al., 2015; Zhuang et al., 2020; Li et al., 2021). To determine if noise exposure alters the profile of microglia in a brain region-specific manner across aging, we carried out immunolabeling of the microglial protein ionized calcium binding adaptor molecule 1 (Iba1) and quantified its expression in the auditory cortex, hippocampus, and striatum using two metrics (Figure 5). First, as microglia transition from a ramified shape with branched processes to a non-ramified amoeboid morphology upon activation (Kettenmann et al., 2011), we characterized the soma-to-cell-size ratio of Iba1 expressing cells. Using this metric, activated microglia with a non-ramified amoeboid shape have a higher ratio than ramified microglia. Second, cell counts in the auditory cortex, hippocampus and striatum determined if the overall density of Iba1-positive cells was affected by noise exposure. Our analysis of the hippocampus focused specifically on the CA1 region given its crucial role in spatial learning performance (Moser et al., 1994; Okada et al., 2003) as well as its implication in mediating age-related spatial learning impairments (Nicholson et al., 2004; Tombaugh et al., 2005; Burger et al., 2007). Separate three-way ANOVAs (treatment × sex × brain region) were conducted for these metrics, ultimately revealing no differences between treatment groups in soma-to-cell-size ratio at any age (Figures 5C–E). However, there was a significant reduction in the number of Iba1 positive cells within the hippocampal CA1 region of noise-exposed rats at 13 months of age [treatment × brain region interaction: *F*_(2,34)_ = 3.730, *p* = 0.034; *p*_bonf_ < 0.05; Figure 5H], with no change in density observed in the other brain regions at any age (Figures 5F,G). Overall, noise exposure altered the profile of microglia expression in the hippocampus at 13 months of age, characterized by a decrease in microglial density; a noise-induced effect that was not observed in the auditory cortex or striatum.

### There were no significant correlations between the degree of hearing loss, spatial learning impairments, and hippocampal microglial density in the 13-month-old rats

**FIGURE 6:**
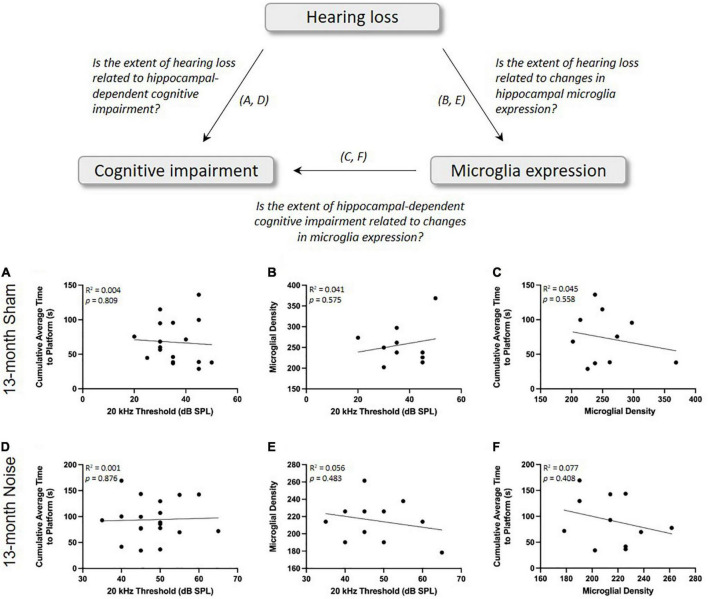
There were no significant correlations between the degree of hearing loss, spatial learning impairments, and hippocampal microglial density in the 13-month-old rats. As determined by a linear regression, the extent of hearing loss was not a significant predictor of spatial learning ability **(A,D)** or hippocampal microglial density **(B,E)** for sham- or noise-exposed rats. **(C,F)** Hippocampal microglial density was also not correlated with spatial learning ability in sham- or noise-exposed rats.

In addition to investigating the effects of noise exposure on cognitive impairment and microglial expression, we were also interested in assessing whether spatial learning deficits or microglial density were directly associated with the degree of hearing loss. Separate linear regressions were performed for sham- and noise-exposed animals at 13 months of age and revealed that hearing loss did not predict the extent of hippocampal-dependent cognitive impairment (Sham: *R*^2^ = 0.004, *p* = 0.809; Noise: *R*^2^ = 0.001, *p* = 0.876; Figures 6A,D) or microglial density (Sham: *R*^2^ = 0.041, *p* = 0.575; Noise: *R*^2^ = 0.056, *p* = 0.483; Figures 6B,E). Furthermore, at the same time point, we sought to explore if spatial learning deficits were dependent upon hippocampal microglial density in the noise- vs. sham-exposed rats. A linear regression revealed no significant relationship between the two variables, indicating that the noise-induced microglial changes did not predict the extent of cognitive impairment (Sham: *R*^2^ = 0.045, *p* = 0.558; Noise: *R*^2^ = 0.077, *p* = 0.408; Figures 6C,F).

## Discussion

### The behavioral consequences of occupational-like noise exposure varied depending on the cognitive domain and time post-noise exposure

In the present study, we investigated if occupational-like noise exposure causes differential deficits in cognitive tasks primarily reliant on the hippocampus vs. striatum, and whether these deficits persist (or become exacerbated) in the months post-exposure. In addition to studying hippocampal-dependent spatial learning using the Morris water maze, we conducted the first investigation into the effects of noise exposure on visuomotor associative learning, a cognitive ability which is known to rely on striatal function. Based on our past work that found a noise-induced deficit in stimulus-response habit-learning (Wieczerzak et al., 2021), we predicted that noise exposure would also cause impairments in a more complex visuomotor associative learning task. While there were no noise-induced deficits in performance accuracy on this striatal-dependent task in any age group, we did observe an unexpected decrease in reaction time (i.e., faster responses) immediately post-exposure in the 7-month-old rats; a finding that may be related to altered dopaminergic signaling in the striatum. Support for this suggestion is derived from a past study which found a direct relationship between faster reaction times in rats trained on a lever-pressing task and an increased number of D_2_ dopamine receptors in their striatum (Macrae et al., 1988), as well as previous studies that reported increased striatal dopamine levels following an occupational-like noise exposure (Ravindran et al., 2005; Samson et al., 2006). Given that the noise-induced decrease in reaction time was short-lived (i.e., it was not observed in the 10- and 13-month-old rats), it would be worthwhile for future studies to examine the interplay between noise exposure, reaction time and dopamine signaling in the striatum. Finally, we suggest that our finding of faster reaction times in noise-exposed rats performing a *visual*-based associative learning task could have implications for *auditory*-based tasks that rely on reaction time as a metric of noise-induced hypersensitivity to sound (Radziwon et al., 2019). Indeed, due to potential alterations in striatal dopaminergic signaling, noise-exposed rats may show faster reaction times regardless of the sensory modality being tested, thereby calling into question the utility of reaction time as a specific measure of altered auditory processing post-noise exposure.

In contrast to the lack of impairment in visuomotor associative learning, the occupational-like noise exposure led to deficits in spatial learning that emerged in an age-dependent manner, such that 13-month-old rats demonstrated impaired performance on the Morris water maze several months after the cessation of the noise exposure. Importantly, these results confirm that noise exposure in early adulthood can indeed exacerbate age-related cognitive decline, as spatial learning was not impaired in the younger 7-month-old rats. As previous studies have reported deficits in spatial learning soon after noise exposure (Cui et al., 2009; Cheng et al., 2011; Chengzhi et al., 2011; Liu et al., 2016; Wang et al., 2016; Wieczerzak et al., 2021), our novel findings highlight the relevance of also considering the protracted consequences of noise exposure on cognition. In the present study, we also investigated whether some rats were more vulnerable to noise-induced cognitive deficits than others, as this could influence their trajectory for age-related cognitive decline. To that end, we performed a quartile-split of the Morris water maze data and observed a deficit in spatial learning in a subset of the 10-month-old rats—those that were already performing poorly compared to their age group (Figure 3F). Thus, it seems that noise exposure was most detrimental to those rats that were prone to cognitive impairment, suggesting that noise exposure may represent an increased risk for age-related deficits in an already-vulnerable group of subjects.

Past studies on humans and animal models have reported that the susceptibility to noise-induced hearing loss can vary considerably amongst individuals, even when exposed to the same noise exposure (Taylor et al., 1965; Mulrow et al., 1990; Seidman and Standring, 2010). With this in mind, we examined if the poorly performing 10-month-old rats experienced a greater degree of noise-induced hearing loss than their better-performing counterparts, and we determined whether the overall results of spatial learning in the 13-month-old noise-exposed rats could be predicted by their hearing thresholds. Ultimately, the poorly performing 10-month-old rats had similar post-exposure 20 kHz hearing thresholds as the good-performers (poor: 35–50 dB SPL; good: 45–80 dB SPL), and we found no relationship between the noise-exposed rats’ 20 kHz hearing thresholds and their spatial learning ability as assessed by the cumulative average time to reach the hidden platform in the Morris water maze (Figure 6D). Collectively, these findings could have significant translational implications as researchers and clinicians would not likely be able to reliably predict who may go on to experience cognitive impairments later in life based simply on the degree of hearing loss they experience due to noise exposure earlier in adulthood.

### Noise exposure in early adulthood caused age-dependent and brain region-specific changes in microglial expression

Microglia have a diverse set of functions within the brain, contributing not only to the neuroinflammatory response during cellular damage, but also helping to maintain homeostasis under resting conditions by regulating synaptic plasticity (Kettenmann et al., 2011). Thus, either excessive activation of microglia or a loss of microglial activity can have devastating effects on neural function. Given their important role, it is not surprising that altered microglial expression has been implicated in mediating aspects of noise-induced pathophysiology within the brain, potentially contributing to auditory and cognitive dysfunction. For example, we and others have previously shown altered microglial expression in the cochlear nucleus following noise exposure, implicating their involvement in noise-induced damage repair and plasticity (Baizer et al., 2015; Fuentes-Santamaria et al., 2017). Similarly, within the auditory cortex, an increase in microglial expression soon after exposure to an intense noise has been proposed as a contributing factor to the aberrant neural plasticity underlying generation of the phantom auditory perception tinnitus (Wang et al., 2019). In contrast, another study using a similar intense noise exposure (Zhuang et al., 2020), as well as the present study using an occupational-like noise exposure, observed no such change in microglial expression in the auditory cortex. Taken together, these findings suggest that the relationship between noise exposure and microglia status can vary across the levels of the central auditory system; however, more research is required to uncover the diverse functions microglia may play in subcortical and cortical regions of the auditory pathway following noise exposure.

While it is well-established that the effects of noise exposure extend beyond the central auditory system, it was unclear if noise-induced changes in microglial expression are brain region-specific, or whether these changes contribute to the profile of cognitive impairment observed post-noise exposure. In the present study, we carried out the first investigation of potential noise-induced alterations in microglial expression within the striatum. In light of the lack of cognitive impairments that we observed in our striatal-dependent visuomotor associative learning task post-noise exposure, it is perhaps not surprising that we did not observe changes in microglial expression within the striatum at any age; results that suggest that the striatum is seemingly resilient to the effects of noise exposure. In contrast, our findings of noise-induced microglial changes in the hippocampus extend the results of previous studies which suggest that this brain region appears to be particularly vulnerable to noise exposure (Cui et al., 2009, 2015; Kraus et al., 2010; Cheng et al., 2011, 2016; Li et al., 2014; Tao et al., 2015; Liu et al., 2016; Wang et al., 2016; Hayes et al., 2019). Most notably, our finding of reduced microglial cell density in the hippocampus of 13-month-old noise-exposed rats is consistent with a previous study which also documented a long-term decrease in microglial cell number, albeit after a much more intense noise exposure (Zhuang et al., 2020). That we observed this alteration in microglial expression long after an occupational-like noise exposure, further emphasizes just how sensitive the hippocampus appears to be to auditory insults. Moreover, the fact that our occupational-like noise exposure and the intense noise exposure used by Zhuang et al. (2020) both caused protracted changes in microglial expression in the hippocampus raises two important questions: (1) Does the degree of hearing loss induced by the noise exposure predict the extent of microglial changes in the hippocampus? and (2) Do these changes in microglial expression contribute to the extent of cognitive impairment?

First, to investigate the potential relationship between hearing loss and the extent of microglial changes in the hippocampus, we performed a linear regression on the 13-month-old rats’ 20 kHz hearing threshold and the cell density of microglia in their CA1 hippocampal region. For both the sham- and noise-exposed rats, we found no significant correlation (Figures 6B,E); findings that were perhaps not too surprising given that, compared to the 13-month-old rats, the 7- and 10-month-old rats showed the same degree of noise-induced hearing loss (Figure 2), yet they did not show any changes in their hippocampal microglial cell density (Figures 5F,G). These time-dependent changes in microglia warrant additional consideration, as ours is the first study to report that it took several months, not just days/weeks, following the noise exposure before the decrease in hippocampal microglial cell density emerged. Second, as past studies have suggested that noise-induced changes in microglial expression cause hippocampal dysfunction (e.g., impaired neurogenesis) (Zhuang et al., 2020; Li et al., 2021), we investigated whether noise-induced changes in microglial expression contributed to the extent of cognitive impairment following our occupational-like noise exposure. To that end, we performed a linear regression between hippocampal microglial density and the 13-month-old rats’ cumulative average time to reach the hidden platform in the Morris water maze. Ultimately, as there was no significant relationship between these variables (see Figures 6C,F), we suggest that the decrease in microglial density did not predict the noise-exposed rats’ impaired spatial learning ability. In further support of this conclusion, we also found that the poorly performing 10-month-old rats did not show a tendency for a decrease in hippocampal microglial density compared to their sham-exposed counterparts.

In considering our collective findings in which reduced hippocampal microglial density was not correlated with the level of hearing loss or performance on the Morris water maze, we suggest that caution be taken when attempting to ascribe noise-induced hearing loss associated deficits in spatial learning to altered microglial expression, as our findings do not support that conclusion. Instead, it is possible that occupational-like noise exposure, regardless of the degree of associated hearing loss, primes the hippocampus for an accelerated age-related phenotype characterized by the co-occurrence of a reduced microglial density and impaired spatial learning. Support for our suggestion of an accelerated age-related phenotype post-noise exposure comes from past studies that have independently documented that aging results in a decline in microglial cell number (both resting and activated microglia) (Hayakawa et al., 2007; Cerbai et al., 2012) along with other molecular changes (Nicholson et al., 2004; Tombaugh et al., 2005; Burger et al., 2007) specifically within the CA1 in aged animal models, as well as age-related impairments in hippocampal-dependent behavioral tasks (Rosenzweig and Barnes, 2003; Shukitt-Hale et al., 2004; Bizon et al., 2009); the two outcomes observed in our 13-month-old noise-exposed rats. In light of our findings, we predict that had we extended the later time-point of our study, we may have observed cognitive impairment and reduced microglial density in the hippocampus in these older sham-exposed rats, thereby confirming that the outcomes in the 13-month-old noise-exposed rats were indeed due to an earlier-onset of an aged phenotype.

### Future directions

The novel findings of the present study not only confirm that there is a complex relationship between noise exposure, hearing loss and cognitive decline, but they also identify worthwhile future directions for preclinical studies. For example, given our finding that a subset of younger animals showed noise-induced deficits in spatial learning, suggesting that noise exposure may represent an increased risk for cognitive impairment in vulnerable subjects, additional studies utilizing animal models of genetic susceptibility to age-related cognitive impairments are warranted (Paciello et al., 2021). Furthermore, in addition to tracking the longer-term consequences of noise exposure on aging animals (i.e., beyond the 13-month time-point investigated in the present study), future studies should also consider investigating the effects of continuous noise exposure on age-related cognitive decline using daily exposures across the lifespan. This approach would more closely resemble the daily noise exposures experienced by individuals working in noisy environments and allow for researchers to investigate whether prolonged noise exposure acts cumulatively to worsen cognition, or whether adaptive mechanisms allow aged animals to compensate through greater reliance on multiple cognitive domains. Related to the possibility of cognitive compensation, it is worth restating that aging is normally associated with an increased reliance on the striatum to help compensate for hippocampal dysfunction (for review, see Zhong and Moffat, 2018). With this in mind, it is reasonable to question whether the differential behavioral effects observed in the noise-exposed 13-month old rats (i.e., impaired spatial learning; unaffected visuomotor associative learning) were the result of an interplay between an age-related reliance on striatal processing coupled with an increased vulnerability of the hippocampus to noise exposure. Ultimately, to further investigate the relationship between noise exposure and cognitive compensation in aging, future studies could investigate noise-induced hippocampal- vs. striatal-dependent cognitive impairment and neuropathology in animals with a genetic susceptibility to age-related cognitive decline.

In addition to the aforementioned studies, researchers could also consider conducting mechanistic studies to better understand the interplay between the intensity and duration of noise exposure, the profile of altered microglial expression, and the presence/absence of cognitive impairment. For example, future studies could combine noise exposures with pharmacological manipulations (e.g., PLX3397) that disrupt the microglia population; an approach that proved successful at revealing how microglia mediate noise-induced plasticity in the auditory cortex (Wang et al., 2019). Ultimately, this mechanistic approach could help to confirm whether noise-induced changes in microglial expression play a causal role in the short- and/or long-term consequences of noise exposure on hippocampal function (e.g., neurogenesis; synaptic plasticity) and cognitive abilities (e.g., spatial learning; reference memory; episodic-like memory). Furthermore, our novel finding that decreased microglia density was not predictive of the extent of hippocampal-dependent cognitive impairment in the noise-exposed rats suggests that other molecular mechanisms likely underlie how noise exposure may accelerate aging in the hippocampus. For example, it is well known that alterations in glutamatergic neurotransmission related to NMDA receptors can result in spatial learning and memory impairments (for review, see Riedel et al., 2003) and that NMDA receptor dysfunction is commonly seen in the brain across aging (Magnusson, 1998; Sonntag et al., 2000; Zhao et al., 2009). Of particular interest, past studies have shown that chronic noise exposure results in decreased levels of the NR2B subunit of the NMDA receptor in the hippocampus (Cui et al., 2009, 2013), suggesting that future studies should explore whether alterations in hippocampal glutamatergic signaling contribute to noise-induced spatial learning deficits across aging.

## Conclusion

In the present study, we investigated the effects of occupational-like noise exposure on cognitive function and microglial expression across aging in a rodent model. Our results highlight that not all higher-level cognitive tasks or their associated brain regions appear to be equally susceptible to noise-induced deficits during aging, with the hippocampus demonstrating greater vulnerability compared to the striatum. Furthermore, our finding of noise-induced cognitive deficits in a subset of younger animals, combined with the apparent lack of a relationship between the degree of hearing loss with cognitive function, highlights the need for future studies to investigate the factors, beyond hearing loss, that make some subjects more susceptible to cognitive dysfunction following noise exposure. Overall, our findings suggest that even a mild occupational-like noise exposure earlier in adulthood can have long lasting implications for cognitive function later in life; a finding with significant clinical implications given the high prevalence of noise exposure across common environmental, recreational, and occupational settings.

## Data availability statement

The raw data supporting the conclusions of this article will be made available by the authors, without undue reservation.

## Ethics statement

This animal study was reviewed and approved by the Animal Care Committee at Western University and in compliance with the guidelines established by the Canadian Council on Animal Care.

## Author contributions

BA, SH, and SW: project conceptualization. SP, CD, SB, SH, and AS: data collection and analysis. SH, BA, SP, and SW: manuscript writing and editing. All authors interpreted the data.
