# Supplementary figures and images for: Noise exposure in early adulthood causes age-dependent and brain region-specific impairments in cognitive function
Source: Front Neurosci. 2022 Oct 13;16:1001686. doi: 10.3389/fnins.2022.1001686 (PMC9606802; doi:10.3389/fnins.2022.1001686)

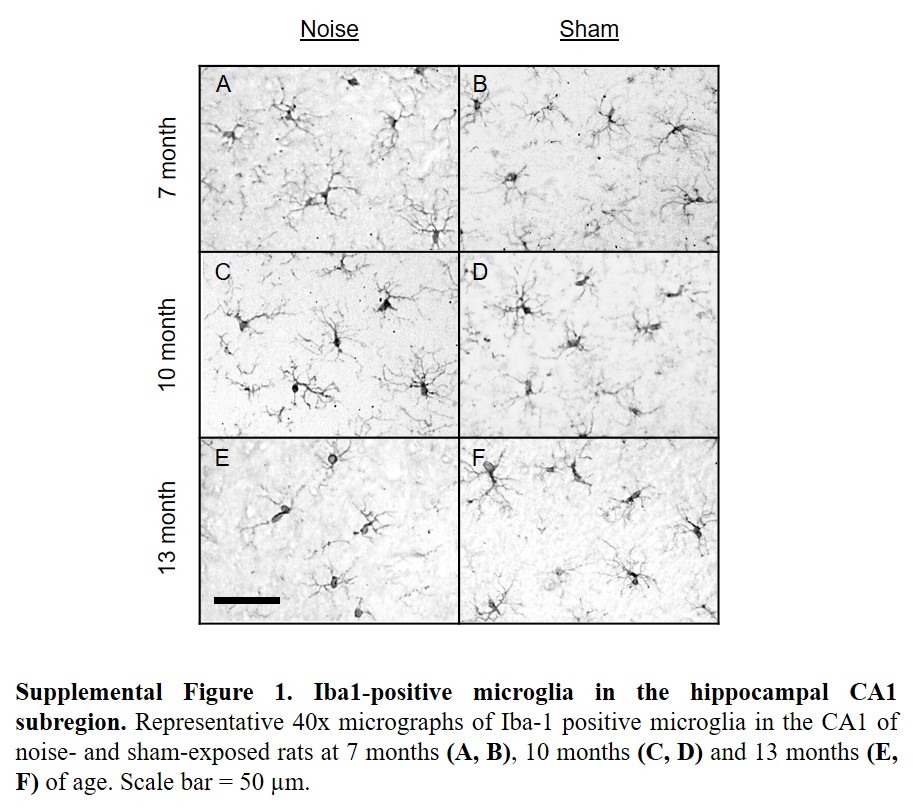

Supplement: Supplementary file 1 [file Image_1.JPEG]
